# Effect of Hay Steaming on the Estimated Precaecal Digestibility of Crude Protein and Selected Amino Acids in Horses

**DOI:** 10.3390/ani12223092

**Published:** 2022-11-10

**Authors:** Caroline Pisch, Monika Wensch-Dorendorf, Uwe Schwarzenbolz, Thomas Henle, Jörg Michael Greef, Annette Zeyner

**Affiliations:** 1 Institute of Agricultural and Nutritional Sciences, Martin-Luther-University Halle-Wittenberg, 06108 Halle (Saale), Germany; 2 Institute of Food Chemistry, Technische Universität Dresden, 01062 Dresden, Germany; 3 Federal Research Center for Cultivated Plants, Crop and Soil Science, Julius Kuehn Institute, 38104 Braunschweig, Germany

**Keywords:** hay, hay steaming, protein, protein solubility, precaecal digestible crude protein, Maillard reaction, equine asthma, horse nutrition, forage nutritive value, horse

## Abstract

**Simple Summary:**

Forage contaminated with pathogenic microorganisms such as bacteria, moulds and yeasts with abiotic deposits represents a serious risk for the health of horses. Due to associated allergens, horses can develop allergic reactions, which may trigger respiratory diseases and would require drastic interventions in feeding regimens and housing conditions. Therefore, horse owners have started to treat hay with steam to achieve a reduction in inhalable allergens. However, the present study has shown that steaming led to the formation of Maillard reaction products, which decreased the proportions of precaecal digestible crude protein and precaecal digestible amino acids. As a consequence, these losses should be considered in ration calculation for horses that receive steam-treated hay.

**Abstract:**

Steaming hay is increasingly used to treat low-quality forage because it was proven to reduce inhalable allergens such as mould spores, bacteria, and airborne dust particles. Preliminary results have shown a substantial loss of precaecal (pc) digestibility (D) of crude protein (CP) and amino acids (AA). For this purpose, six different batches of hay from central Germany were divided into four subsamples, and each one was individually steamed. Native hay and four replicates of each steamed subsample were analysed for CP, AA, neutral detergent insoluble crude protein (NDICP), neutral detergent soluble crude protein (NDSCP) as well as pepsin insoluble CP (piCP). Based on the analytical parameters, pcD of CP, protein solubility (PS), piCP (% CP) and precaecal digestible (pcd) CP and pcdAA contents were calculated. Selected Maillard reaction products (MRP), namely furosine and carboxymethyllysine (CML), were also analysed. Steaming did not affect CP content (native = 69, steamed = 67 g/kg dry matter, DM; *p* > 0.05), but it had an impact on the insoluble part of CP. Thus, NDICP increased by 57% (native = 27, steamed = 42 g/kg DM; *p* < 0.05) and piCP by 15% overall (native = 40, steamed = 46% of CP; *p* < 0.05). This could be a consequence of the heat damage and the associated increase in MRP. The content of furosine rose by 67% (native = 17.6, steamed = 29.4 mg/100 g DM; *p* < 0.05). The content of CML increased by 120% (native = 5.1, steamed = 11.3 mg/100 g DM; *p* < 0.05). We chose to analyse these two MRPs because they represent the reaction products with the limiting AA lysine. In contrast, the soluble fractions of CP declined, while PS as a percentage of CP decreased by 38% as a result of the treatment, and NDSCP was reduced by as much as 41% (*p* < 0.05). In line with this, the steaming process decreased the pcD of CP (native = 56%, steamed = 35%; *p* < 0.05) and pcdCP (native = 37.9, steamed = 22.5 g/kg DM; *p* < 0.05), respectively. The same effects were shown for selected AA; e.g., sulphuric AA pcd methionine plus pcd cysteine decreased by 45%, pcd threonine decreased by 41%, and the limited AA pcd lysine decreased by more than 50% (*p* < 0.05). In conclusion, the high temperatures generated during steaming lead to protein damage and consequently to a reduction in the pcD of CP and essential AA. Nevertheless, steaming successfully reduces viable microorganisms and binds dust particles. Therefore, steamed hay is still a proper and sometimes the only possible roughage for horses suffering from respiratory diseases such as equine asthma. Essentially, horse diets based on steamed hay should be balanced accordingly.

## 1. Introduction

In horse husbandry, it is a prerequisite to provide feed, especially forage, and embedding materials of high hygienic quality to ensure optimal nutrient supply and the prevention of respiratory airway diseases [1,2,3]. Due to a strong regional diversity in terms of soil, growth and climate circumstances, there are severe differences in the nutrient content of hay [4,5]. Different weather conditions during the conservation process and insufficient storage conditions significantly affect the hygienic quality of the feed [2,6]. Feeding hygienically inadequate hay or housing on poor straw supports the pathogenesis of chronic inflammatory respiratory disease [7,8,9]. The disease is considered to be non-infectious and allergic. Historically, it has been described as chronic obstructive pulmonary disease (COPD), small airway disease, or recurrent airway obstruction (RAO), and it is also known as “heaves” or inflammatory airway disease (IAD) [9,10]. As the disease shares several attributes with human asthma, including pathophysiological aspects and clinical presentation, the preferred term is currently equine asthma (EA) [10]. It can be divided into mild/moderate (IAD) and severe (RAO) forms [8,11,12]. The mild and moderate form is the most common one with a total prevalence of circa 60% of the adult horse population worldwide [13]. Severe asthma is estimated to affect 10–15% of adult horses [12,13,14]. Today, approximately 50% of the 12–14-year-old horses housed in stables already suffer from EA in Germany [15].

Treating hay with hot steam is a highly reliable method established to reduce the viability of adherent microorganisms and to bind airborne dust with a success rate up to 99% [16,17,18]. Thus, this method shows good success for forage optimisation and is a long-term approach for heaves-affected horses [19]. The hay should be steamed for one hour, and a temperature of approximately 100 °C should be reached in the feed. Steamed hay was stable during a test period up to 24 h when stored at 10 or 25 °C, respectively [17]. This is particularly interesting regarding the possibility to prepare larger amounts of steamed hay for horses in advance. However, the authors investigated only one hay sample and showed a decrease in the precaecal (pc) digestibility (D) of crude protein (CP). It has been suggested that the pcD of CP is reduced due to heat damage and thus has detrimental effects on forage quality from a nutritional point of view [17].

The aim of the present study was to determine whether steaming increases the insoluble parts of CP and the formation of Maillard reaction products (MRPs), which would indicate the heat damage of feed protein. We hypothesised that the high temperatures generated during steaming result in a measurable amount of protein damage and consequently reduce the pcD of CP and essential amino acids (EAA), which should be considered especially for broodmares, growing horses and sport horses.

## 2. Materials and Methods

### 2.1. Study Design

**Figure 1:**
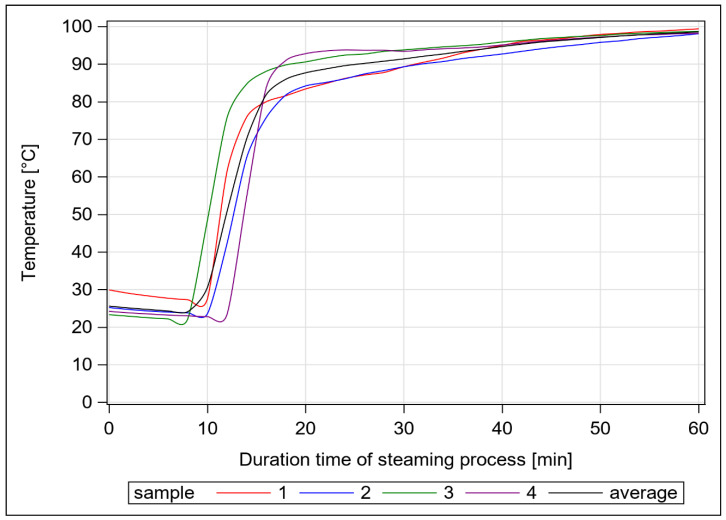
Rise of temperature inside the tested hay nets during steaming in a Haygain One. Specifications are given in the text.

Samples were taken from six different batches of regionally produced hay, which is commonly fed to horses. These hay samples were harvested in 2020 at first cut and at the end of blossom from the typical grass-dominated meadows of Saxony-Anhalt and Saxony. Each sample comprised 16 kg of material, which was divided into four subsamples with four kg hay in nets each. In advance of steaming, a native sample of 350 g (i.e., untreated control) was set aside. According to the manufacturer’s specifications (Haygain, HG One+ for 8 kg; Farm & Stable, West Sussex, UK), the hay nets were steamed for one hour. By the end of the steaming process, a temperature indicator in the lid of the device showed an average of about 97 ± 1.1 °C. Additionally, to detect the temperature inside the hay nets, we placed a special data logger (PCE-HTD 125 data logger; PCE Instruments, Meschede, Germany) with a measurement range from −40 to 125 °C in the centre of four randomly selected hay samples. After about 8 to 10 min, there was a sudden increase in temperature, and after an average of 20 min, the temperature reached 80 °C or more. In our samples, we almost reached 100 °C after 60 min (Figure 1). Immediately after steaming, four replicates with an estimated weight of 350 g each were taken from different locations of the hay net. Subsequently, the samples were oven-dried at 40 °C for two days. The air-dried samples were coarsely pre-grounded and then ground to pass a 1 mm sieve of a laboratory sample mill (Tecator Cyclotech 093; Foss GmbH, Hamburg, Germany). For the analyses of mono- and dimeric sugars, fructans, macro elements, trace elements, amino acids (AA) and MRP, the materials were pulverised using a ball mill (Mixer Mill MM 400; Retsch GmbH, Haan, Germany).

### 2.2. Sensory Analyses

The hays’ sensory properties (i.e., appearance, texture and smell) as well as the hygienic status were determined in a standardised manner following the procedure described by Kamphues et al. [1] (Table S1). Impurities were detected by eye or using a loupe [3]. Individual points were summed up, and total points ranging between 0 and 20 were assigned for forage value and between 0 and −40 for hygienic status (Table S2).

### 2.3. Analytical Methods and Calculations

The concentrations of dry matter (DM), crude ash (CA), crude protein (CP), crude fat (CL), crude fibre (CF) and the van Soest detergent fibres were determined according to the official German Key Book for feed analysis (VDLUFA 2012; methods no. 3.1, 4.1.1, 5.1.1 B. 6.1.1, 6.5.1, 6.5.2, 6.5.3, 8.1) [20] with a FOSS 2300 Kjeltec analyser for nitrogen. Neutral detergent fiber (aNDFom) was assayed using thermostable amylase. Neutral and acid detergent fiber (ADFom) were expressed exclusive of residual ash [21]. Neutral detergent insoluble CP (NDICP) was determined in line with Licitra et al. [22] according to VDLUFA method no. 4.13.1 [23] and used to calculate the neutral detergent-soluble and precaecal digestible (pcd) contents of dietary CP and AA, respectively [24,25,26]. The protein solubility (PS) was calculated as PS = NDSCP × 100/CP, and the precaecal digestibility (pcD) of CP was calculated as pcD = pcdCP × 100/CP [26], with CP and pcdCP given in grams per kilogram of DM. Pepsin-insoluble CP (piCP) originates from protein evaluation for ruminants and is the proportion of CP that is not dissolved by treatment with HCl and pepsin. It was expressed both in absolute terms and as a percentage of CP and analysed according to VDLUFA method 4.2.1 [20]. In brief, 0.15 M HCl solution was prewarmed in a water bath up to 40 °C. Pepsin (0.7 FIP U/mg; MERCK) from pig stomach mucosa was added and dissolved. A quantity of 0.5 g of sample material was weighed into a 300 mL conical flask, and 100 mL of pepsin solution was added. For about 3 min, the solution was stirred on a magnetic stirrer and then incubated at 39 °C for another 16 h period. After incubation, 5 mL of 25% HCl was added. The entire mixture was filtered through an ash-free paper filter and rinsed with approximately 150 mL of distilled water. The filters were stored at room temperature for about 24 h. Subsequently, nitrogen was determined. Nitrogen was generally analysed on a FOSS 2300 Kjeltec (FOSS GmbH, Hamburg, Germany) according to VDLUFA method no. 4.1.1 (Kjeldahl method) [20]. All analyses were performed in triplicate. The feeds’ proteins were hydrolysed with hydrochloric acid and AA were analysed using a Biochrom 30 Amino Acid Analyser with a PEEK Sodium Prewash Column (100 × 4.6 mm) and PEEK-Oxidised Feedstuff Column (200 × 4.6 mm) (Biochrom Ltd., Cambridge, UK) according to VDLUFA method no. 4.11.1 [20]. For the analyses of tryptophan (Trp), proteins were hydrolysed with phosphoric acid and hydrochloric acid, and Trp was analysed using high-performance liquid chromatography (HPLC) and an Agilent 1100 Series unit with ZORBAX Eclipse XDB-C8 (150 × 4.6 mm, 5 μm; Agilent Technologies Inc., Santa Clara, CA, USA) according to Fontaine et al. [27]. The measurements were performed in duplicates.

Selected Maillard reaction products were determined as follows: The amount of furosine as a precursor in the early stage Maillard reaction was quantified by reversed phase-HPLC after acid hydrolysis with 6 N HCl as reported by Krause et al. [28] with separation on a cation exchange resin column and post-column derivatisation with ninhydrin [29]. The Amadori product fructoselysine was calculated from the furosine concentration by multiplying with the transfer factor 3.1 [28,30]. Carboxymethyllysine (CML) represents a lysine-associated product of the final stage of the Maillard reaction. Carboxymethyllysine (CML) represents a lysine-associated product of the final stage of the Maillard reaction. The concentration of CML was analysed after enzymatic digestion by liquid chromatography-mass spectrometry (LC-MS) according to Schwarzenbolz et al. [31]. All measurements were performed in duplicates.

The metabolisable energy (ME) concentration in hay was calculated based on crude nutrient analyses as recommended by the German Society of Nutrition Physiology (GfE) according to Kienzle and Zeyner [24,25]. In addition, the ME content of the hay was determined considering dynamic renal energy losses according to Kuchler et al. [32].

Macro- and trace elements were analysed by inductively coupled plasma optical emission spectrometry (Varian 715-ES ICP-OES) following extraction as described by Rodehutscord and Dieckmann [33].

The water-soluble carbohydrates (glucose, fructose and sucrose) were analysed by HPLC on a KONTRON Instruments unit (Tresser Instruments, Rossdorf, Germany) with a refractive index detector (Shodex RI-71; Showa Denko Europe GmbH, Shodex Business, Munich, Germany) and 100 mm × 7.8 mm separation column (Rezex RPM-Monosaccharide Pb+2; Phenomenex Ltd. Deutschland, Aschaffenburg, Germany) [34]. Fructan were analysed via the chromatographic method according to Pavis et al. [35].

### 2.4. Statistical Analysis

Statistical analysis was performed using the SAS 9.4 software package (SAS Institute Inc., Cary, NC, USA). Least square means (LSM) were estimated and compared using the following mixed linear model and the MIXED procedure:

*y*_ij_ = *μ* + *α*_i_ + *B*_j_ + *ε*_ij_

in which *y*_ij_ are nutrients and energy contents, *μ* is the general mean, *α*_i_ is the fixed effect of treatment *i* (*i* = 0, 0, where 0 = native hay and 1 = steamed hay), *B*_j_ is the random effect of batch *j* (*j* = 1,…, 6) and *ε*_ij_ is the random residual effect. Additionally, a linear regression approach was used to assess linear relationships between lysine to furosine, pcdLys to furosine, pcD of CP to piCP, pcD of CP to lysine as well as PS to furosine and PS to CML. Differences with *p* < 0.05 were considered to be significant.

## 3. Results

### 3.1. Sensory Analyses

The hays used in this study were of sensory and nutritional quality typical for Central Germany conditions. The sensory assessments are summarised in Tables S1 and S2.

The first hay was macroscopically of good quality but slightly dusty. Hay two was rather straw-like, had a musty smell, was sandy, and was very dusty. The third hay showed a good forage quality but was heavily occupied with rodent droppings. Hay four was also very straw-like, contained many plants with a poor feed value, e.g., sorrel and many foreign seeds. Hay five and six were macroscopically of good quality but were slightly dusty and showed a slight musty odour (Table 1).

### 3.2. Proximate Nutrients

**Figure 2:**
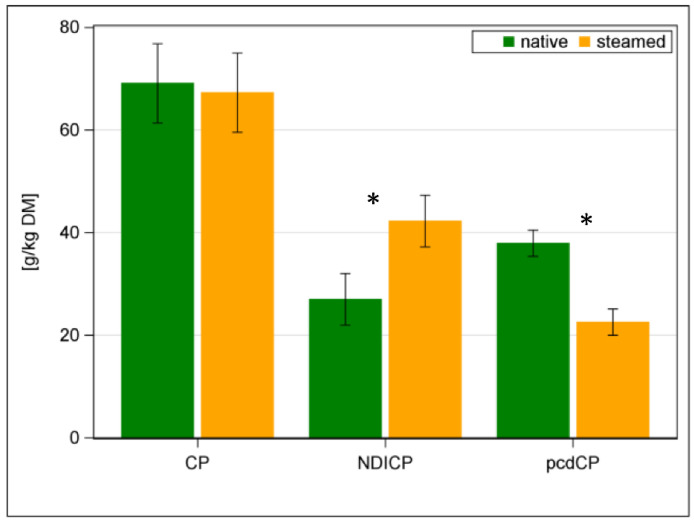
Contents of crude protein (CP), neutral detergent insoluble CP (NDICP) and precaecal digestible CP (pcdCP) in native (green bars) and steamed hay (orange bars); * with *p* < 0.05 significant differences of means.

The native hay had a DM content of 961 g/kg, whereas it was reduced by steaming to 888 g/kg (*p* < 0.001). Contents of CA, CP, CF and ADFom were similar in the native and steamed hays (Table 2). The aNDFom content increased after steaming (*p* < 0.05; Table 2). This was also applicable to the ADL content (Table 2). Contents of WSC (i.e., glucose + fructose + sucrose) of the original hays ranged from 79 to 146 g/kg DM (data not shown). The overall effects of steaming hay on WSC content were variable and not significant. The WSC content of hays 1, 2, 3 and 5 decreased by 8, 1, 3 and 4%, respectively. The WSC content of hays 4 and 6 increased by 5 and 12%, respectively (data not shown). Similarly, the ME content did not differ between native and steamed hay (Table 2). Following steaming, there was a significant shift within the CP, and the proportion of NDICP increased by 57% on average. Thereby, the content of pcdCP decreased by 41% (Figure 2; *p* < 0.001; Table 2). This was also reflected in a reduction in pcD of CP, from native with 56% to steamed hay with 35% (*p* < 0.001; Table 2).

**Figure 3:**
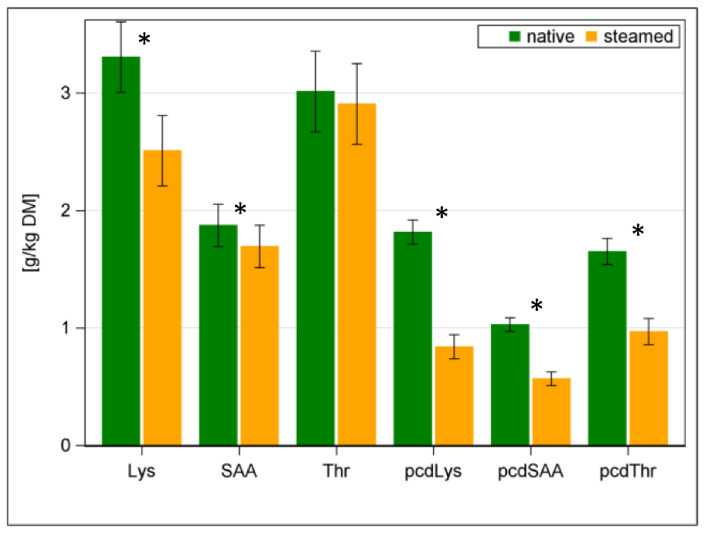
Contents of selected essential amino acids (EAA) and precaecal digestible EAA (pcdEAA) in native (green bars) and steamed hay (orange bars); * with *p* < 0.05 significant differences of means; Lys = lysine, SAA = sulphuric amino acids (i.e., methionine + cysteine), Thr = threonine.

Pepsin-insoluble CP as a percent of CP resulting from heat damage by drying, ensiling, pelleting process or in this case, by steaming, experienced an average increase of about 15% (*p* < 0.001). In line with the reduction in pcD of CP, the AA and pcdAA are also decreasing (Figure 3; Table 3). The bar graph in Figure 3 presents the original content of selected EAA on the left side. Thereby, lysine (Lys) decreased from native to steamed hay by 24% and the sulphuric AA (SAA) as a sum of methionine (Met) and cysteine (Cys) was reduced by 10% (*p* < 0.001). Only threonine did not decrease significantly. The right side of Figure 3 shows the content of the pcdAA. The pcdLys content decreased by 54%, and the other pcdAA declined by more than 40% in total (pcdSAA was reduced by 45%; pcdThr was reduced by 41%; *p* < 0.001; Table 3).

### 3.3. Maillard Reaction Products

**Figure 4:**
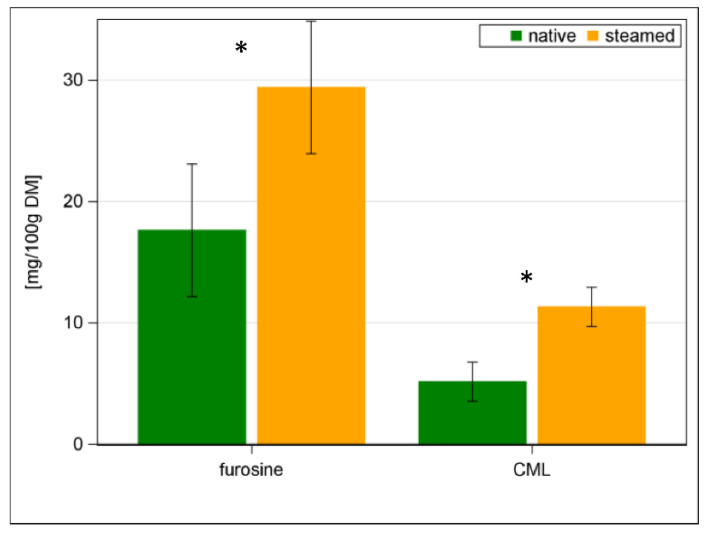
Maillard reaction products in native and steamed hay (* with *p* < 0.05 significant differences of means).

**Figure 5:**
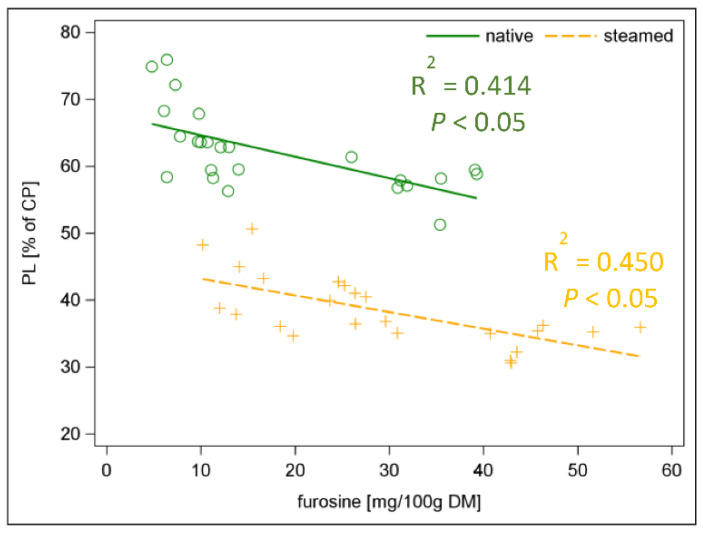
Linear relationship between protein solubility (percentage of CP) and furosine (mg/100 g DM) in native and steamed hay.

**Figure 6:**
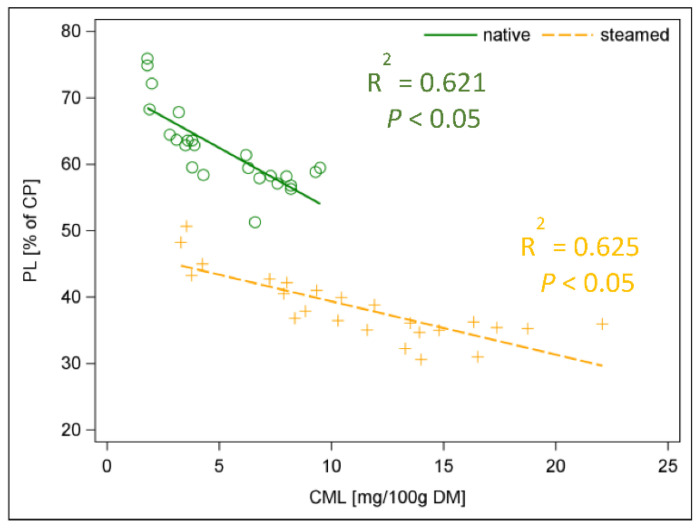
Linear relationship between protein solubility (% CP) and CML (mg/100 g DM) in native and steamed hay; CML = carboxymethyllysine.

As a result of steaming, the early stage MRP furosine increased by 67% and the final stage MRP CML increased by 120% (Figure 4; *p <* 0.001). If one relates the limited AA Lys with the MRP furosine, a negative relationship can be found (*R*^2^ = 0.59) (Figure S1). In addition, the pcdLys content had a strong negative relationship to furosine (*R*^2^ = 0.74) (Figure S2, Table S3). In food research and industry, furosine offers the advantage of being a direct marker for the formation of Lys-related MRPs [36]. This was confirmed by our results, but it is quite difficult to analyse these MRPs. Therefore, we were looking at what the correlation between pcD of CP and furosine looks like (Figure S3) and if piCP as a percentage of CP could be an alternative. The pcD of CP was negatively correlated to furosine (*R*^2^ = 0.83) as well as pcD of CP to piCP (as percentage of CP) (*R*^2^ = 0.84) (Figure S4). Both relationships were significant for each individual batch (Table S3). In addition, the PS was related to the analysed MRPs both in native and steamed hays (Figure 5 and Figure 6). The native hay showed higher PS than steamed hay. A negative correlation between PS and MRP was observed (*R*^2^ = 0.41 and *R*^2^ = 0.45 for furosine and *R*^2^ = 0.62 and *R*^2^ = 0.63 for CML in native and steamed hay, respectively) (Figure 5 and Figure 6). The formation of MRPs thus started even at low temperatures during hay making.

The calcium (Ca) concentration was slightly higher in the native hays compared with the treated ones (*p* < 0.05), whereas phosphorus (P) concentration was nearly equal (Table 2). The magnesium (Mg) concentration was lower in the steamed hay (Table 2). The treatment effect on all trace elements was negligible (Table 2).

## 4. Discussion

According to the sensory analyses protocol of Kamphues et al. [1], all hay samples showed small deficits in the hygienic status and also in the nutritional value. Most of them were dusty or musty and partly contaminated with rodent droppings (clear deficits). Using the system of Kamphues et al. [1], the forage value has to be judged critically, since it evaluates feed with a high leaf content to have high nutritional quality and rewards high-fibre forage with only a few points. The high leaf content of the present hays indicated high nutritive value, but horses do also need a high-fibre content in the diet. On the one hand, this is particularly important for the innate need to chew and thus reduces stereotypic behaviours such as crib biting, wood chewing or wind sucking [37,38]. On the other hand, a sufficiently long chewing time, which a high-fibre diet supports, increases alkaline saliva production and buffers acids in the stomach [37,39].

The nutrient contents of the six meadow hay samples examined in this study are listed in Table 1. These represent typical regional hay varieties for feeding horses [17,21]. That steaming hay reduces the abundance of adhering microorganisms, moulds, spores and bacteria by around 99% has been shown many times [16,17,18,40]. Blackman et al. [41] as well as Moore-Colyer et al. [16] have also shown that steaming hay conserves the minerals, trace elements and CP content, which is in line with the results of this study (Table 2). However, a large shift within the CP from the soluble fraction of the cell content to the insoluble fraction bound to the cell wall was observed. The NDICP, which has increased probably due to heat damage, cannot be broken down by the enzymes of the small intestine, making the protein nutritionally unavailable to the organism. The large shift within CP must be considered and balanced in the requirement calculation for horses fed steamed hay because it has a direct negative effect on pcD of CP and AA and thus on the content of pcdCP and pcdAA. According to the feed evaluation system recommended by the GfE [25], no differences in ME concentration of untreated and treated hay appeared due to the similar crude nutrient contents. This was clearly different for pcdCP, pcdAA as well as pcD of CP and AA. The GfE recommends that a maintenance pcdCP requirement of 3 g/kg body weight^0.75^ has to be covered. That means a 600 kg warmblood-type horse should receive 365 g pcdCP daily. The native hay in the present study had a pcdCP content of 38 g/kg DM, which decreased by 41% to 23 g/kg DM in the steamed hay. Therefore, a 600 kg horse fed a hay diet only would require approximately 10 kg/d DM of native hay but 16 kg/d DM of steamed hay to meet its daily pcdCP requirement. That would mean an increase of 180 kg per month DM. Moreover, pcD of CP and AA (56% in native hay) was significantly reduced by 38% points (to 35%) after steaming. On the other hand, studies on the voluntary food intake of horses were able to show that the intake of steamed hay was higher than that of dry hay [42,43]. The large loss of pcdEAA during steaming should not be ignored. The formation of large amounts of MRPs during the steaming process might be an explanation for the reduced pcD of CP and EAA as well as decreasing pcdCP and pcdAA contents. Van Soest [44] described changes in the cell wall composition due to heat treatment of feed as well as insufficient digestion of lignin-like Maillard polymers. Despite the low digestibility of the protein-bound product, further studies are necessary to clarify the bioavailability of MRP [31,45]. However, it has already been shown that colon microbes in particular are able to metabolise MRP [45,46,47]. We choose to analyse furosine and CML because this reaction occurs primarily at the α-amino group of free lysine residues or on the epsilon amino group of terminal peptide- or protein-bound AA, particularly at the lysine side chains of the proteins [48]. Furosine, as an early stage product of the Maillard reaction, can be used as an indicator of the extent and progress of the reaction and thus to evaluate lysine blockade [36]. However, we have also found a reduction in other AAs that are potential reactants for the Maillard reaction, e.g., arginine, tryptophan, histidine and cysteine side chains of proteins and peptides [49]. The strong decreases of AA, pcdEAA as well as pcdCP, with a simultaneous increase in MRPs, confirmed this effect. This is extremely important. Horses can only absorb AA in the small intestine, but the AA transporters are not able to transport glycated AA [45]. According to GfE recommendations for maintenance, a 600 kg horse should receive 15 g pcdLys daily [25]. Steaming reduced pcdLys by 54% from 1.82 g/kg DM in native to 0.84 g/kg DM in steamed hay. It would therefore require an additional intake of pcdLys of approximately 117% to meet the requirements for the important limiting AA lysine. Specifically, this means that for a 600 kg horse, the owner must steam a portion of approximately 17 kg/d DM hay (=117%) to avoid undersupply of CP and AA when feeding steamed hay. Especially for broodmares, growing horses and sport horses, the reduction in pcD of CP and AA as well as pcdCP and pcdAA must be considered and compensated [25]. Nevertheless, it should not be ignored that although the requirement for steamed hay to meet pcdCP and pcdLys increased significantly, the ME content did not vary as a result of the treatment. Consequently, the resulting elevated hay intake could lead to an oversupply of energy.

In order to assess protein damage by treatment, it could be an easier way to analyse piCP instead of MRPs. Linear regression between pcD of CP and furosine and between pcD of CP and piCP revealed an equal negative relationship. The analysis of piCP could therefore be an adequate alternative to that of specific MRPs and offers the possibility of estimating the proportion of insoluble CP that is indigestible due to heat damage.

The total WSC contents of the hay determined in this study were within the values that are typical for the region of Saxony and Saxony-Anhalt [17,21]. Steaming did not affect WSC contents to a relevant extent. Since the WSCs in native hay were already above 100 g/kg DM and were not reduced by steaming, the treatment method is not recommended for horses with metabolic disorders or laminitis-prone equids [21,50,51]. Looking to the future, the increasing energy costs in all areas should not be forgotten. The steaming process should be completed in one hour. The steam generator used in this study requires 1.5 kWh, and it was possible to treat a hay net weighing about 4 kg at a time. If a 600 kg horse is fed this hay with an ME content of 6.9 MJ/kg DM, it would need about 9 kg/d DM of hay to meet its energy requirements. However, as described above, this is not sufficient to meet the pcdCP requirement of a hay-only diet. To meet the requirement via hay, 10 kg/d DM of native and 16 kg/d DM of steamed hay would have to be provided. Using the same device as in this study, it would take about 6 kWh per day to provide sufficient steamed hay. That means an additional 180 kWh in one month.

## 5. Conclusions

Respiratory airway diseases, in particular the pathogenesis of equine asthma, are a massive problem in horse husbandry today. Since most triggering factors are inhalable allergens, horse owners are increasingly used to treat hygienically low-quality hay with hot steam to eliminate the viable microorganisms. The results of this study revealed that steaming elevates the insoluble part of CP with serious consequences in terms of decreasing pcD of CP and AA and thus decreasing the contents of pcdCP and pcdAA. This detrimental effect can partly be attributed to heat damage. This has been indicated by increasing MRPs following steaming. However, steaming hay is still a practical method to treat hygienically inadequate hay and sometimes provides the only possible roughage for horses suffering from equine asthma. A horse’s diet based on steamed hay should be appropriately balanced with particular emphasis on pcdEAA.

## Figures and Tables

**Table 1:** Results for sensory analysis of each hay sample. Sensory analysis were performed according to Kamphues et al. [1].

Hay Sample	Forage Value	Points	Hygienic Status	Points
Hay 1	satisfying (dusty)	13	flawless	0
Hay 2	very low (straw-like, dusty)	3	clear deficits (musty, sandy)	−8
Hay 3	satisfying	15	clear deficits (rodent droppings)	−7
Hay 4	moderate (straw-like)	6	small deficits	−2
Hay 5	satisfying	10	small deficits	−5
Hay 6	satisfying	11	small deficits	−5

**Table 2:** Least square means and standard error (SE) of analysed nutrients, including calculated contents of pcdCP, pcD of CP, pcdEAA and metabolisable energy depending on the treatment (native or steamed).

Item	Unit	Treatment			
		Native	Steamed	SE	*p*-Value
DM	g/kg	961	888	±3.4	<0.001
CA	g/kg DM	62	61	±3.7	0.551
CP	g/kg DM	69	67	±7.7	0.29
CL	g/kg DM	8	9	±0.8	0.352
CF	g/kg DM	330	334	±15.2	0.411
aNDFom	g/kg DM	673	695	±18.5	<0.001
ADFom	g/kg DM	371	372	±14.9	0.869
ADL	g/kg DM	49	52	±3.2	0.003
NDICP	g/kg DM	27	42	±5.0	<0.001
NDSCP	g/kg DM	42	25	±2.8	<0.001
pcdCP ^a^	g/kg DM	38	23	±2.6	<0.001
piCP	g/kg DM	27	31	±3.4	<0.001
pcD of CP ^a^	% of CP	56	35	±2.0	<0.001
PS ^a^	% of CP	62	38	±2.3	<0.001
piCP	% of CP	40	46	±1.3	<0.001
glucose	g/kg DM	13	11	±1.7	0.001
fructose	g/kg DM	25	25	±3.2	0.838
sucrose	g/kg DM	9	11	±1.9	0.009
fructans	g/kg DM	65	67	±9.3	0.22
total WSC	g/kg DM	113	113	±12.7	0.709
CML	mg/100 g DM	5.1	11.3	±1.61	<0.001
furosine	mg/100 g DM	17.6	29.4	±5.46	<0.001
P	g/kg DM	1.84	1.83	±0.139	0.878
Ca	g/kg DM	3.99	3.66	±0.317	0.011
K	g/kg DM	13.51	13.68	±2.214	0.634
Na	g/kg DM	0.09	0.07	±0.044	0.108
Mg	g/kg DM	1.37	1.31	±0.126	0.034
Zn	mg/kg DM	38.65	39.37	±13.159	0.493
Mn	mg/kg DM	114.06	120.53	±45.260	0.06
Cu	mg/kg DM	4.54	4.58	±0.572	0.766
Fe	mg/kg DM	142.73	180.1	±34.178	0.061
ME ^b^	MJ/kg DM	6.9	6.9	±0.3	0.648
ME mod. ^c^	MJ/kg DM	6.8	6.8	±0.3	0.599

DM, dry matter; CA, crude ash; CP, crude protein; CL, crude fat; CF, crude fibre; aNDFom, neutral detergent fibre treated with a heat-stable amylase and expressed exclusive of residual ash; ADFom, acid detergent fibre, expressed exclusive of residual ash; ADL, acid detergent lignin; ME, metabolisable energy; NDICP, neutral-detergent insoluble CP; NDSCP, neutral-detergent soluble CP; pcdCP, precaecal digestible CP; pcD of CP, precaecal digestibility of CP; PS, protein solubility; piCP, pepsin-insoluble CP; total WSC, water soluble carbohydrates in sum of glucose, fructose, sucrose, fructans; CML, carboxymethyllysine; ^a^ calculated according to GfE [25] and Zeyner et al. [26], ^b^ calculated according to Kienzle and Zeyner [24] and GfE [25]. ^c^ calculated according to Kuchler et al. [32].

**Table 3:** Least square means and standard error (SE) of the analysed contents of essential and non-essential amino acids (AA) in meadow hay depending on treatment (native or steamed).

	Treatment of Hay							
	Native		Steamed		SE		*p*-Value	
	g/kg DM	g/16 g N	g/kg DM	g/16 g N	g/kg DM	g/16 g N	g/kg DM	g/16 g N
Essential amino acids								
Histidine	1.1	1.6	1	1.4	±0.12	±0.06	<0.001	<0.001
Isoleucine	2.9	4.1	2.8	4.1	±0.34	±0.10	0.367	0.393
Leucine	5.2	7.6	5.1	7.4	±0.61	±0.17	0.189	0.229
Lysine	3.3	4.8	2.5	3.7	±0.30	±0.10	<0.001	<0.001
Methionine	1.2	1.7	1.1	1.6	±0.13	±0.04	0.003	0.011
Phenylalanine	3.2	4.6	3.1	4.5	±0.41	±0.14	0.133	0.085
Threonine	3	4.4	2.9	4.3	±0.34	±0.08	0.155	0.176
Tryptophan	0.9	1.3	0.9	1.3	±0.12	±0.05	0.047	0.007
Valine	3.6	5.3	3.5	5.2	±0.43	±0.10	0.253	0.263
Non-essential amino acids								
Alanine	4.3	6.2	4.1	6	±0.51	±0.14	0.133	0.147
Arginine	3.5	5	3.2	4.6	±0.38	±0.09	<0.001	<0.001
Aspartic acid	6.1	8.7	5.8	8.4	±0.74	±0.20	0.091	0.034
Cysteine	0.7	1.1	0.6	0.9	±0.05	±0.05	<0.001	<0.001
Glutamic acid	7.1	10.2	6.8	9.9	±0.82	±0.17	0.112	0.09
Glycine	3.6	5.2	3.5	5.1	±0.41	±0.09	0.228	0.307
Proline	4	5.6	3.9	5.5	±0.68	±0.41	0.622	0.525
Serine	2.9	4.2	2.8	4.1	±0.32	±0.07	0.069	0.074
Tyrosine	2.1	3	1.9	2.8	±0.22	±0.08	0.006	0.005
Precaecal digestible AA ^a^								
pcdLys	1.82		0.84		±0.103		<0.001	
pcdMet	0.63		0.36		±0.043		<0.001	
pcdCys	0.4		0.21		±0.018		<0.001	
pcdSAA	1.03		0.57		±0.058		<0.001	
pcdThr	1.65		0.97		±0.112		<0.001	

pcdLys, precaecal digestible lysine; pcdMet, precaecal digestible methionine; pcdCys, precaecal digestible cysteine; pcdSAA, precaecal digestible sulphuric amino acids; pcdThr, precaecal digestible threonine; ^a^ calculated according to GfE [25] and Zeyner et al. [26].

## Data Availability

All data are included in this article.

## Supplementary Materials

The following are available online at https://www.mdpi.com/article/10.3390/ani12223092/s1, Table S1: Evaluation of the hygienic status of hay by sensory analysis in accordance with Kamphues et al. [1]. Table S2: Evaluation of forage value and hygienic status according to Kamphues et al. [1]. Figure S1: Lysine [g/kg DM] compared to furosine [mg/100 g DM]. Figure S2: pcdLys [g/kg DM] compared to furosine [mg/100 g DM]. Figure S3: pcD of CP in [% CP] compared to furosine [mg/100 g DM]. Figure S4: pcD of CP in [% CP] compared to piCP in [% CP]. Table S3: Linear regression for individual batch of hay with *R*^2^ and significance *p*-value.
