# Supplementary material for: Effect of Hay Steaming on the Estimated Precaecal Digestibility of Crude Protein and Selected Amino Acids in Horses
Source: Animals (Basel). 2022 Nov 10;12(22):3092. doi: 10.3390/ani12223092 (PMC9686632; doi:10.3390/ani12223092)
Supplement: Supplementary file 1 [file animals-12-03092-s001.zip › animals-1998454-supplementary.pdf]

Table S1. Evaluation of the hygienic status of hay by sensory analysis in accordance with Kamphues et al. [1].

Parameters	nutritional value		hygienic status	
	findings	points deducted	findings	points deducted
Texture	soft and leafy ((a lot) inflorescence)<br>low-leaved<br>very low leaved<br>stem-rich (inflorescences)<br>strawy (deflowered)	10<br>7<br>5<br>2<br>0	dry<br>slightly clammy<br>clammy, moist	0<br>-2<br>-5
Odor	aromatic (typically)<br>lightly aromatic / mouldy<br>flat / rotten	3<br>1<br>0	unremarkable<br>musty nuances<br>mouldy / putrid	0<br>-5<br>-10
Colour	nicely green<br>slightly discoloured<br>strongly discoloured	5<br>3<br>1	product - typical<br>focally grey, whitish<br>diffusely discoloured	0<br>-2<br>-5
Impurities	macroscopic free<br>low sand/ soil content<br>high sand/ soil content (turf, root mass, etc.)	2<br>1<br>0	none<br>medium grade<br>high grade	0<br>-5<br>-10
Note:	possibly evaluate the proportion of plants with low forage value (thistles/honeysuckle)	-5 to -10	Containing waste material. poisonous plants, plant parts altered by disease	-5 to -10

Table S2. Evaluation of forage value and hygienic status according to Kamphues et al. [1].

nutritional value	points	hygienic status	points
very good	20 – 16	flawless	0
satisfying	15 – 10	small deficits	-1 to -5
moderate	9 – 5	clear deficits	-6 to -10
very low (strawy)	4 – 0	massive deficits	-11 to -40

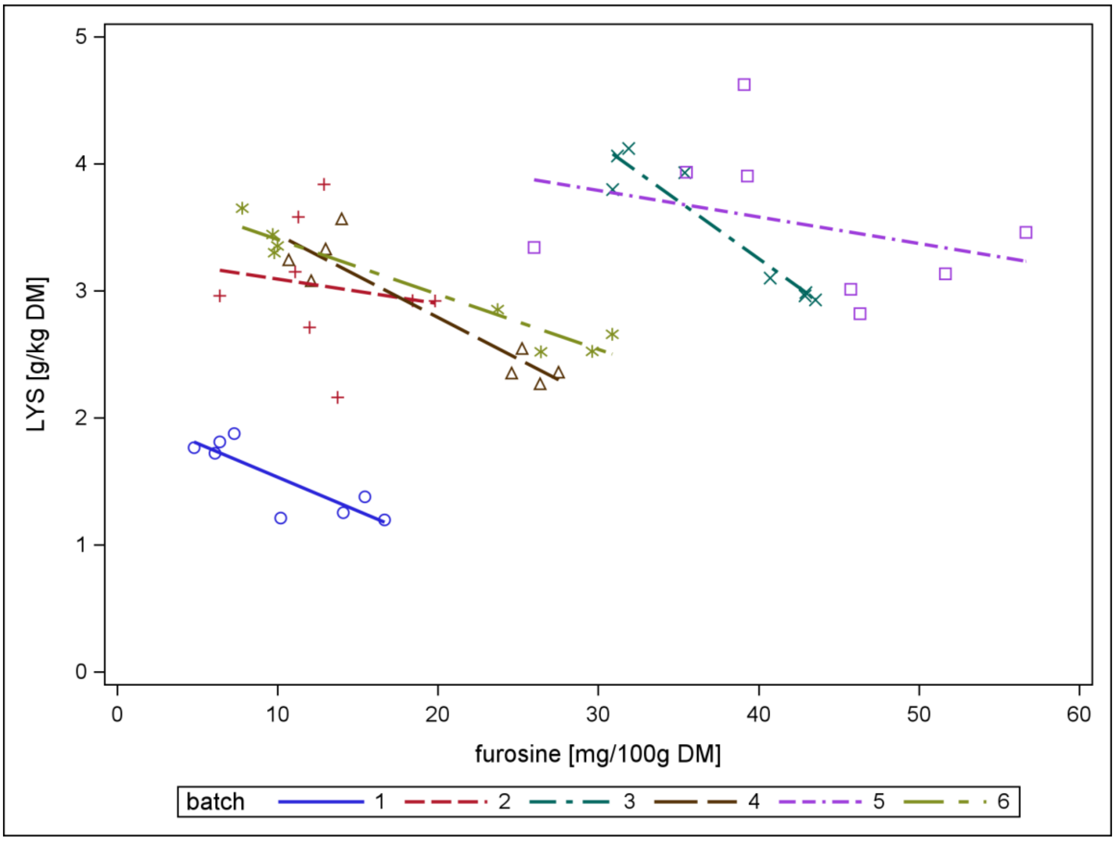

Figure S1. Lysine [g/kg DM] compared to furosine [mg/100g DM]

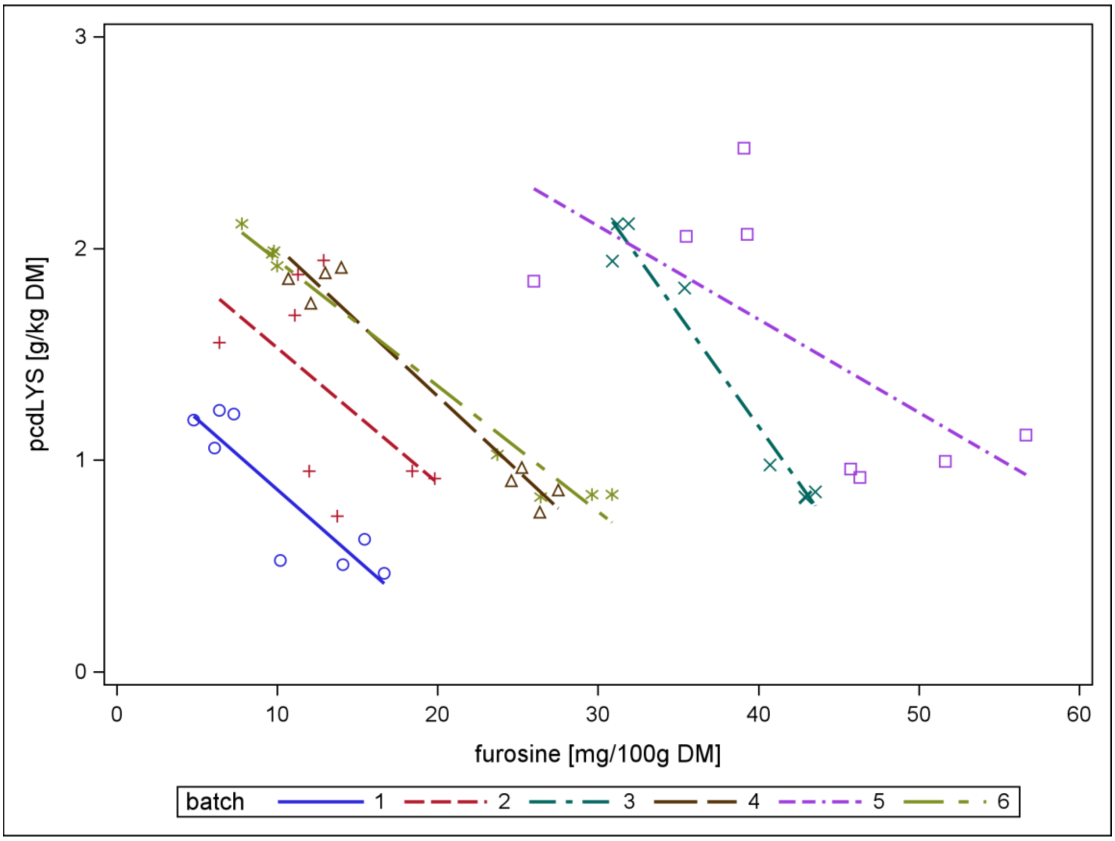

Figure S2. pcdLys [g/kg DM] compared to furosine [mg/100g DM]

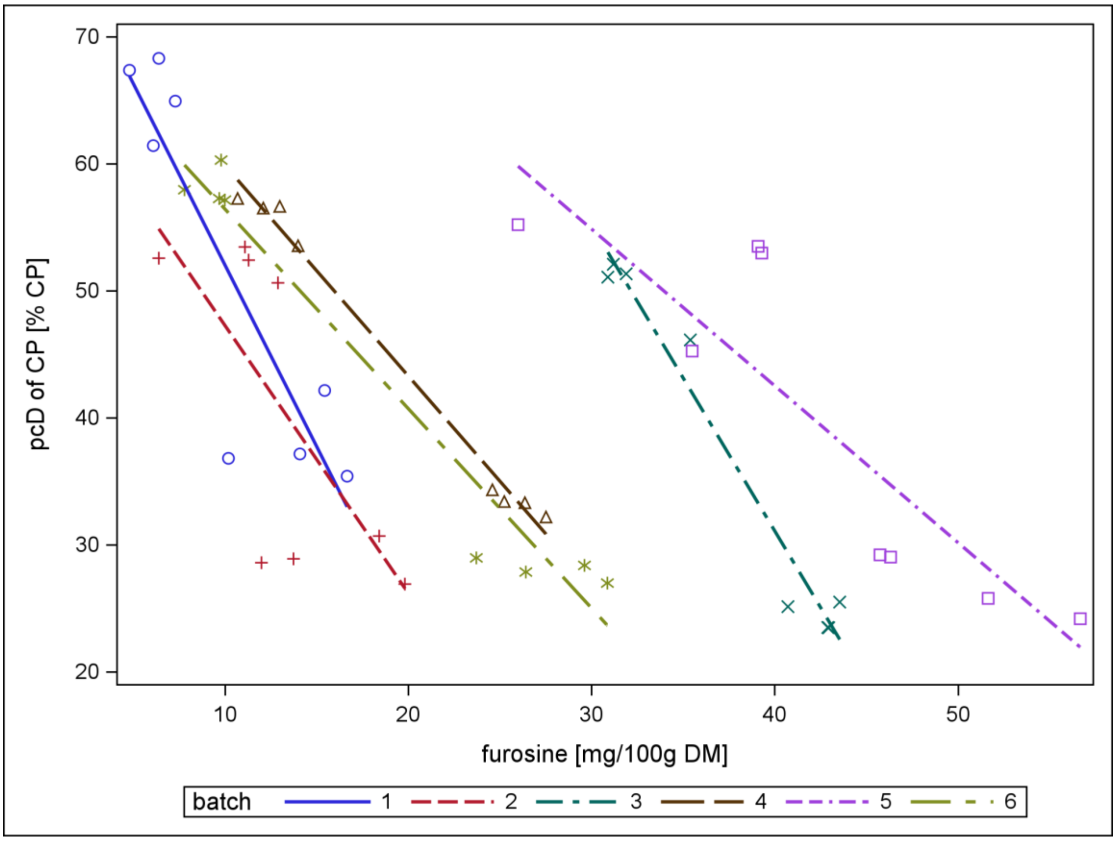

Figure S3. pcD of CP in [% CP] compared to furosine [mg/100g DM]

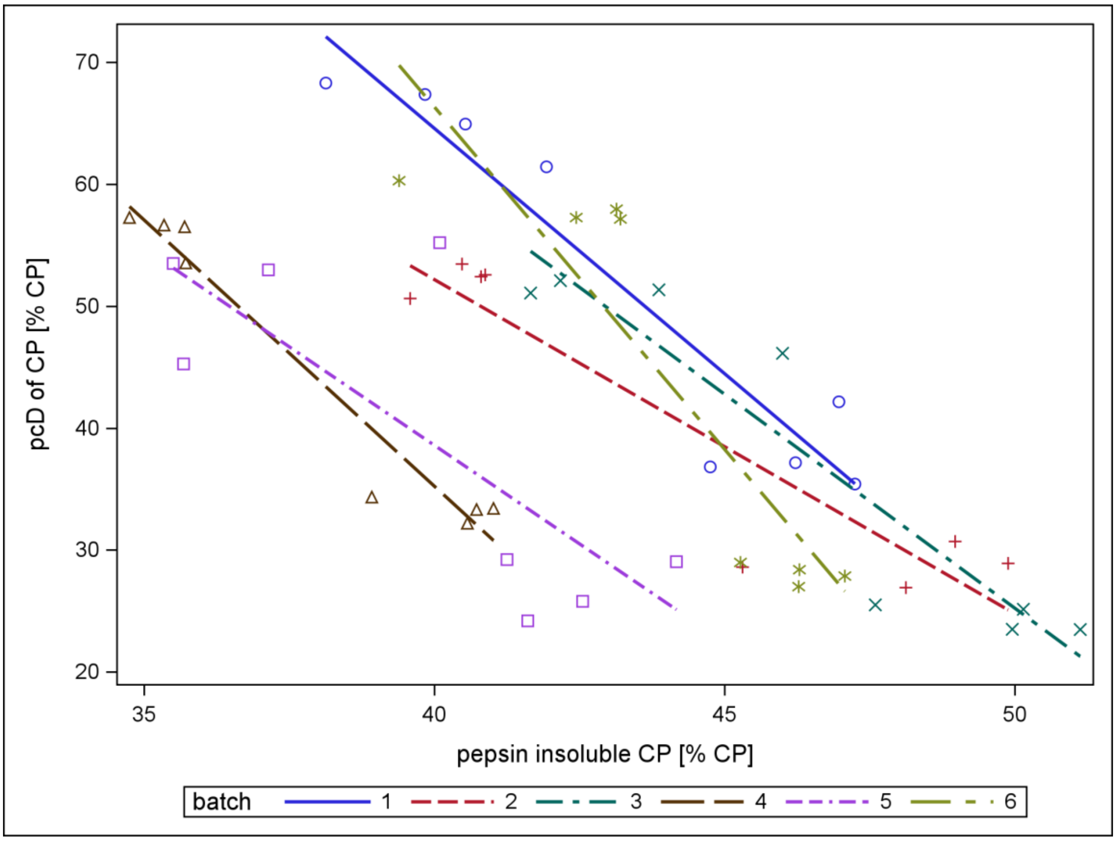

Figure S4. pcD of CP in [% CP] compared to piCP in [% CP]

Table S3. Linear regression for individual batch of hay with R^2 and significance *p*-value

x	furosine		furosine		pcD of CP		pcD of CP	
y	lys		pcdLYS		piCP		furosine	
batch	R^2	*p*	R^2	*p*	R^2	*p*	R^2	*p*
1	0.7199	0.008	0.7884	0.003	0.8997	<0.001	0.7919	0.003
2	0.0255	0.706	0.3104	0.151	0.8722	0.001	0.5072	0.048
3	0.9142	<0.001	0.9663	<0.001	0.885	<0.001	0.9652	<0.001
4	0.854	0.001	0.9538	<0.001	0.9533	<0.001	0.9897	<0.001
5	0.1155	0.410	0.4676	0.062	0.6023	0.024	0.7686	0.004
6	0.9322	0.001	0.9781	<0.001	0.8028	0.003	0.957	<0.001
mean	0.59		0.74		0.84		0.83	

x, x-axis; y, y-axis; Lys, lysine [g/kg DM]; furosine [mg/100g DM]; pcdLYS, pre-caecal digestible lysine [g/kg DM]; pcD of CP, pre-caecal digestibility of CP [% CP]; piCP, pepsin insoluble CP [% CP]
